# Binge-eating behaviors in adolescents and young adults during the COVID-19 pandemic

**DOI:** 10.1186/s40337-022-00650-6

**Published:** 2022-08-24

**Authors:** Melissa Freizinger, Grace B. Jhe, Suzanne E. Dahlberg, Emily Pluhar, Amanda Raffoul, Wallis Slater, Lydia A. Shrier

**Affiliations:** 1 Division of Adolescent and Young Adult Medicine, Boston Children’s Hospital, 300 Longwood Ave, Boston, MA 02115 USA; 2 Department of Psychiatry, Harvard Medical School, 25 Shattuck Street, Boston, MA 02115 USA; 3 Department of Pediatrics, Harvard Medical School, 25 Shattuck Street, Boston, MA 02115 USA; 4 Institutional Centers for Clinical and Translational Research, Boston Children’s Hospital, 300 Longwood Ave, Boston, MA 02115 USA; 5 Division of Adolescent and Young Adult Medicine, Boston Children’s Hospital, 333 Longwood Avenue, 5th Floor, Boston, MA USA

**Keywords:** Adolescents, Binge eating disorder, Bingeing behaviors, COVID-19 pandemic, Eating disorders, Food availability, Food affordability, Young adults

## Abstract

**Background:**

The COVID-19 pandemic and subsequent public health measures have resulted in a worsening of eating disorder symptoms and an increase in psychological distress. The present study examined symptoms and behaviors in adolescents and young adults with emotional eating, bingeing behaviors and binge eating disorder during the pandemic. Additionally, the study explored if individuals who experienced pandemic-related food availability and food affordability issues experienced increased binge-eating symptoms and negative feelings.

**Method:**

Participants (n = 39) were a convenience sample who participated between November 2020 and January 2021 in a weight and lifestyle management program at an urban New England pediatric hospital. Participants completed online surveys that assessed (1) participant’s exposure to COVID-19 related stress and binge-eating behaviors using the COVID-19 Exposure and Family Impact Survey-Adolescent and Young Adult Version (CEFIS-AYA) and the Binge Eating Scale (BES) respectively, (2) participants’ and their families’ ability to attain and afford food and its association with bingeing behaviors, and (3) the relationship between food availability and affordability and negative emotions.

**Results:**

Nearly half of all participants (48.7%) reported moderate to severe bingeing during the COVID-19 pandemic; those who experienced greater COVID-related stress reported more binge-eating behaviors (*p* = 0.03). There were no associations between indicators of food availability and affordability and binge eating or between food availability and affordability and negative feelings.

**Conclusions:**

Higher pandemic-related stress was associated with more binge-eating behaviors among adolescents and young adults. These results underscore the need to monitor symptoms and provide treatment for these patients despite barriers to care imposed by the COVID-19 pandemic. Research and clinical care for adolescents and young adults with EDs must recognize and respond to pandemic effects across the weight and disordered eating spectrum.

## Plain English summary

Research shows that the COVID-19 pandemic continues to have far-reaching adverse effects on mental health. For adolescents and young adults, the COVID-19 pandemic has altered critical aspects of their daily lives. The objective of this study is to investigate binge-eating behaviors in adolescents and young adults during the COVID-19 pandemic and to examine if individuals in households that experienced pandemic-related challenges such as food availability and food affordability had greater increases in bingeing behaviors and negative emotions such as feelings of anxiety, worry, mood, and loneliness. Thirty-nine adolescents and young adults previously assessed in an outpatient weight and lifestyle management program at an urban pediatric hospital were surveyed between November 2020 and January 2021. Almost half (48.7%) of these participants reported moderate to severe bingeing behaviors during the pandemic. Participants who reported higher impact of COVID-related stress on the CEFIS-AYA scale reported the highest level of binge-eating behaviors. There were no associations between food availability and affordability and binge eating or between food availability and affordability and negative feelings. This study highlights the importance of assessing patients’ perception of how they experience the myriad impacts of COVID-19 on their daily lives, and the critical need for increases in accessible mental health services and continued support during the on-going pandemic.

## Background

The COVID-19 pandemic has profoundly affected the epidemiology of eating disorders (EDs), particularly for adolescents and young adults. The initial lockdown period of the COVID-19 pandemic interfered with the recovery trajectory of patients with EDs, as some recovered patients experienced a return of binge eating and compensatory exercise [1]. Additionally, adolescents and young adults with EDs experienced an increase in symptoms, and those with past EDs were at risk for relapse during the pandemic [2, 3]. In one study, almost half of the children and adolescents with EDs experienced a return of ED behaviors, and those with severe EDs experienced symptoms of self-harm and increased suicide risk. [3]

Binge eating disorder (BED) is the most common ED, with lifetime prevalence of 2.2% for adults [4] and 1.32% for adolescents [5]. When sub-clinical binge behaviors are considered, the prevalence is estimated at 3% [5]. There have been numerous studies examining disordered eating and binge-eating behaviors in adults during the pandemic [2, 6–8]. Adults with BED reported increases in binge eating and urges to binge, and many binged on “stockpiled” food [2]. Among adults with a history of BED, binge-eating frequency increased during the pandemic, placing them at risk for relapse [6]. Increases of binge eating have also been found among the general population [8]. However, research on binge-eating behaviors among adolescents and young adults during the COVID-19 pandemic is limited.

Additionally, the COVID-19 pandemic has generated an economic crisis and resulting food insecurity, which is defined as an interruption or inability to access food because of lack of finances [9–11]. Food insecurity has been found to be associated with greater likelihood of binge eating in emerging adults and bariatric surgery candidates. [12, 13] Researchers have noted that the combination of the COVID-19 pandemic and food insecurity may exacerbate EDs and binge-eating behaviors [14]. Individuals with food insecurity are more likely to have a diagnosis of BED [15] and experience more frequent binge eating than those without food insecurity [16]. In a recent study of American university students during COVID-19, students with food insecurity experienced significantly greater binge eating and ED-related impairment than students without food insecurity [16]. Food insecurity has also been linked to poor mental health outcomes such as mood disorders in adolescents and young adults [17, 18]. Emerging data indicates that food insecurity and food worry during the COVID-19 pandemic were associated with increased risk of depression and anxiety symptoms along with poor mental health [19–21]. Little is known about the associations between pandemic-related food issues such as food availability and food affordability, negative emotions, and binge-eating behaviors in adolescents and young adults already engaged in care for binge eating.

Accordingly, the purpose of this study was to investigate the associations between COVID-related stress on binge-eating behaviors among treatment-seeking adolescents and young adults. This study also examined the associations between pandemic-related food challenges such as food availability and affordability and binge-eating behaviors, as well as associations between food availability and affordability and feelings of anxiety, worry, mood, and loneliness among adolescents and young adults in clinical care for BED, emotional eating, and disordered eating behaviors.

## Methods

### Study design

We recruited individuals from a multidisciplinary outpatient weight and lifestyle management program at an urban pediatric hospital in the northeastern United States. This clinic serves patients who are referred by a healthcare provider or independently present for evaluation and treatment for BED, emotional eating, or lifestyle counseling for weight concerns and/or preparation for bariatric surgery.

All individuals between the ages of 13–26 years who were evaluated and/or treated from March 2019 to March 2020 were eligible. Only patients seen in the year prior to the onset of the COVID-19 pandemic were eligible as the goal was to evaluate active patient symptomatology during the initial lockdown period of the COVID-19 pandemic. We identified 70 patients who would comprise the sampling frame for the current study.

Eligible participants (*n* = 70) received an email from the research team introducing the purpose of the study, staff contact information, and an option to continue in or opt out of the study. Participants who responded expressing interest (*n* = 48, 69%) were provided with a link to a one-time survey in RedCap, an online database used to distribute surveys. The survey was preceded by an information sheet describing study rationale, procedures, and potential risks. Continuing with the survey constituted informed consent. Upon survey completion, subjects received a $15 Amazon gift card. Surveys were completed from November 2020 to January 2021; the final sample consisted of 39 respondents who provided complete data. The study was approved by the Hospital Institutional Review Board.

### Outcome measures

#### Demographic features

Patient information, age, and DSM-5 diagnosis were retrieved from clinic data using medical record numbers based on the timeframe of treatment. Data were de-identified and stored in an electronic database for analysis. A brief self-report form queried participant characteristics such as race, gender, education status (i.e., in-school full-time or part-time, on school break or summer vacation, not currently in school), and work status (i.e., working full or part-time, not currently working).

#### Binge eating scale (BES)

Binge eating was assessed using the BES [22], a 16-item self-administered questionnaire containing eight items that describe behaviors (e.g., eating fast or consuming large amounts of food) and eight items on feelings and cognitions (e.g., fear of not stopping eating). Each item has a response range from 0 to 3 (0 = no problem, 3 = serious problem). Overall scores were classified into one of three categories: (a) no binge eating (≤ 17); (b) mild-moderate binge eating (18–26); and (c) severe binge eating (≥ 27). [22, 23]

#### COVID-19 exposure and family impact survey-adolescent and young adult version (CEFIS-AYA)

CEFIS-AYA is a 44-item measure which asks participants to reflect on experiences related to the COVID-19 pandemic and is comprised of two scales assessing exposure and impact [24]. The 28-item (yes/no) Exposure Scale is calculated by totaling the number of affirmative responses that measure “exposure” to COVID-19 related events, including disruption in day-to-day life, difficulty accessing resources, financial stressors, and family exposure to COVID-19. Cronbach’s alpha for the Exposure scale was excellent (α = 0.80). The 16-item Impact Scale assesses the impact of COVID-19 across numerous domains, including physical (e.g., eating and sleeping) and emotional wellbeing. Fifteen of the Impact Scale items use a four-point Likert scale assessing how the pandemic has affected various facets of daily life (1 = Made it a lot better, 4 = Made it a lot worse), and 1 item uses a 10-point distress scale, where higher scores denote higher distress. Four Impact Scale questions were used to measure participant’s negative feelings: anxiety, worry, loneliness and distress. Total CEFIS-AYA scores range from 15 to 98. Cronbach’s alpha for the Impact scale was excellent (α = 0.92).

#### Food availability and affordability

An ad hoc questionnaire was developed using three items from the demographic form and one item from the CEFIS-AYA. Using the demographic form, participants were questioned on difficulty affording groceries (“Did your family have difficulty affording groceries during the ‘stay at home advisory’ phase of COVID-19 pandemic?”; yes/no/sometimes), food availability (“How available was food in your house during the ‘stay at home advisory’ phase of the COVID-19 pandemic?; same/more/less) and worry about not having enough food (“Did you feel worried that you or your family would not have enough food during the ‘stay at home advisory’ phase of the COVID-19 pandemic?; yes/no), and from the CEFIS-AYA Exposure Scale, difficulty getting food (“I/we had difficulty getting food; yes/no).

### Statistical analyses

Given the limited sample size, a power calculation was conducted prior to analyses. Using a two-sided 0.05 level analysis of variance (Kruskal–Wallis) test and assuming that 30 patients were to be enrolled and equally distributed across the three binge-eating severity groups, this study had 83% power to detect an association between the binge eating and CEFIS-AYA impact and exposure scales if the mean CEFIS-AYA scores across the mild, moderate, severe BES groups were 20, 40, and 60, respectively, with a standard deviation of 25 points. In a linear regression model, this power was maintained to detect a change in the regression coefficient for COVID-related stress as a predictor of BES.

Demographics were characterized with descriptive statistics. Categorical data were compared using Fisher’s exact tests for associations between each of the categorical four negative emotion outcomes (anxiety/worry, mood, loneliness, distress) and each of the four different categorical food measures (difficulty affording groceries, food available in the house, worried about not having enough food in the house, and difficulty getting food). Between-group comparisons on continuous outcomes were assessed through the Kruskal–Wallis test, with post hoc pairwise comparison using the Wilcoxon test. If univariate associations were significant, backwards stepwise regression was used for multivariable model fitting with selection for entry into the possible model at the 2-sided 0.20 level; criterion to remain in the final model was a 2-sided *p* < 0.10.

The primary analyses included all registered patients who completed the surveys (*N* = 39). No adjustments were made for multiple comparisons and all *p*-values are 2-sided. Data were analyzed with R, version 3.6.1 [25].

## Results

### Sample demographics

Sample demographics are summarized in Table 1. Of note, there were no statistically significant differences between those who completed the survey (n = 39) and those who did not (n = 31) in terms of their gender and primary diagnosis. Fisher’s exact test was used to test for association between gender (*p* = 0.70) and primary diagnosis (*p* = 0.33). However, age was significantly different between those who participated in the study and those who did not, with a median (IQR) equal to 19 (18–21) for those included in the study and 17 (15–18.5) for those not included in the study (*p* = 0.001).

**Table 1:** Sociodemographic characteristics of adolescents and young adults (N = 39)

*Age*	
Mean (SD)	19.3 (3.2)
*Race/ethnicity*	
Asian	1 (2.6)
Black of African American	11 (28.2)
Hispanic or Latinx	9 (23.1)
White	18 (46.2)
*Gender*	
Female	31 (79.5)
Male	6 (15.4)
Non-binary	1 (2.6)
Prefer to self-describe	1 (2.6)
*Diagnosis*	
Binge eating disorder	22 (56.4)
Emotional or mindless eating	8 (20.5)
Evaluation for bariatric surgery	6 (15.8)
Other	3 (7.6)
*Education*	
Graduated school	2 (5.1)
In school full-time	16 (41.0)
In school part-time	6 (15.4)
Not currently in school	11 (28.2)
On school break	4 (10.3)

### COVID-19 impact and binge-eating symptoms

**Fig. 1:**
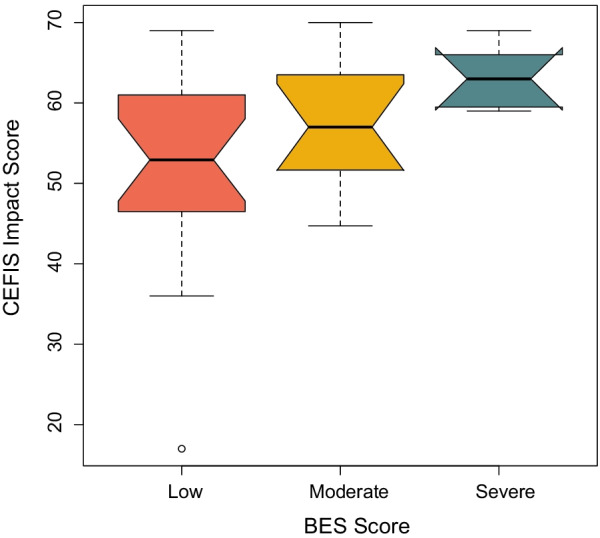
CEFIS-IMPACT score by BES group

CEFIS-AYA Impact scores significantly differed by BES groups (Kruskal–Wallis chi-squared = 6.76, df = 2, *p* = 0.03). The median of impact scores was 52.9 (range = 17–69) among non-binge eaters (n = 20), 57 (range = 44.7–70) among moderate binge eaters (n = 12), and 63 (range = 59–69) among severe binge eaters (n = 7). A multivariable linear regression model was fitted to try to ascertain whether the association between (continuous) BES and impact scores was maintained after adjusting for other prognostic factors. Impact scores were significantly associated with higher BES scores (F(2, 36) R2 = 0.23; beta (SE) = 0.27 (0.13), *p* = 0.04; see Table 2), suggesting that greater COVID-related stress was associated with more binge-eating behaviors. Figure 1 illustrates the differences in CEFIS-IMPACT score by BES group.

**Table 2:** Linear regression summary table: Association between COVID-related stress and binge-eating behaviors

Variable	*β*	SE (*β)*	*p*-value
CEFIS IMPACT	0.2740	0.1318	0.04
Age	− 1.0660	0.4407	0.02

There was no association between CEFIS-AYA Exposure Score and BES score in a linear regression model of continuous BES (F(1, 37) *p* = 0.33; R2 = 0.03; beta (SE) = 0.37 (0.37)).

### Food availability and affordability, binge-eating behaviors, and negative emotions

There was no association between the level of food availability and affordability and binge-eating behaviors. Although there was a moderate univariate association between difficulty affording groceries and BES total score, this was not maintained in a multivariable model after attempting to adjust for age, race, and gender (F(1, 37) *p* = 0.098; R2 = 0.07; beta (SE) =  − 5.31(3.13)). There was also no association between food availability in the home (F(1, 37) *p* = 0.97; R2 =  < 0.001; beta (SE) =  − 0.18 (4.12), worriedness about having sufficient food in the home (F(1, 37) *p* = 0.40; R2 = 0.02; beta (SE) =  − 2.87 (3.39), nor difficulty getting food (F(1, 37) *p* = 0.84; R2 = 0.001; beta (SE) = − 0.92 (4.37)) and BES total score. Lastly, we did not observe association between negative feelings (emotional well-being, depression, loneliness, distress) and food availability and affordability.

## Discussion

This study provides additional information regarding adolescents and young adults with binge-eating behaviors and BED during the COVID-19 pandemic. Nearly half of the participants (48.7%) reported an increase in bingeing behaviors during the COVID-19 pandemic. Of note, those individuals who reported greater impact from the COVID-19 pandemic also endorsed more binge-eating symptoms, while COVID-19 pandemic exposures were not associated with binge-eating behaviors. The findings indicate that the perception of how the pandemic has affected various domains of life (e.g., physical and emotional well-being) may have contributed to binge-eating behaviors, not merely the presence of stressors (e.g., financial stressors, limited access to resources).

The association between the impact of the COVID-19 pandemic and study participants experiencing binge-eating symptoms in this study is consistent with literature. [1, 2, 6], Many individuals experienced increased binge eating, overeating, and using food to cope during the pandemic [8]. This occurrence is likely due to the relationship between higher psychological distress and a subsequent higher risk of eating disturbances [26]. It is further possible that the pandemic has exacerbated individuals’ experiences of stress and isolation, which are related to increases in emotional eating and overeating [27]. In fact, the social isolation of the COVID-19 pandemic may not only exacerbate ED symptoms, but also increase anxiety and stress for those with EDs [28]. Difficulties with emotion regulation and a lack of coping skills during times of heightened stress are known to trigger binge-eating episodes [29]. Our findings suggests that those patients who feel the COVID-19 pandemic has negatively impacted their lives across multiple areas and report the pandemic has made their daily life worse are at risk of exhibiting greater binge-eating symptoms. Future research is needed to further examine the role of the COVID-19 pandemic and increased binge eating in the context of stress levels and pre-existing coping skills.

A secondary aim of this study was to examine if adolescents and young adults who had pandemic-related food issues (i.e., food availability, food affordability, worries about having enough food, difficulty with getting food) experienced increased binge-eating behaviors. We did not find significant associations between indicators of food availability and affordability and binge-eating symptoms. This finding is inconsistent with previous research that found the COVID-19 pandemic contributed to an increase in bingeing behavior due to factors such as increased food insecurity and shortages [30, 31]. Although individuals may have experienced increased pandemic-related food challenges, the current finding suggests that the experience of food availability and affordability alone may not be associated with increased binge-eating behaviors in our population. It may be that our sample was too small to show effects, or that our younger-aged participants were unaware of food issues in their households. Research has been inconsistent regarding the association between food insecurity and disordered eating among adolescents [32], these associations may be more robust in adults [33]. Although a recent longitudinal study of adolescents followed into young adulthood found associations between severe food insecurity and disordered eating, the strongest associations were for extreme weight control behaviors (self-induced vomiting, diet pill use), with binge-eating behaviors emerging 5 years later [33]. Additionally, our study used single items for food availability and affordability rather than a validated tool that measured the full construct of food insecurity, which may have impacted the results.

The present study also did not find any significant association between COVID-related food availability and affordability and negative feelings. As noted, it may be that our sample was not directly impacted by or aware of food challenges in their household. Research indicates that parents try to protect their children from the effects of household level food insecurity with younger children being protected to a greater extent than older children [34]. Additionally, some data suggest that not all individuals experienced the pandemic as negative [35–38]. In one study, participants identified that social distancing reduced social comparison, where others found increased time with family a deterrent to ED behaviors [31]. In another study, some participants reported that the COVID-19 lockdown was a “catalyst” for increased motivation for recovery [35]. Some of our participants may have experienced increased support and less social anxiety by being at home during the COVID-19 pandemic. Indeed, a recent study found that some adolescents experienced decreased levels of stress related to social domains and their depressive symptoms remained stable, however, they did experience an increase in maladaptive behaviors such as bingeing [39]. The role of COVID-related negative feelings and food availability and affordability in adolescents and young adults and potential protective factors need to be further examined in a larger sample.

To our knowledge, this is one of the few studies to examine the associations between pandemic related impacts and exposures and binge-eating behaviors in a cohort of adolescents and young adults, many of whom were diagnosed with BED. Limitations of this study should be noted. This was a small sample, although power calculations did show sufficient power for our primary outcomes. Additionally, surveys were self-reported and thus subject to reporting bias. Due to the self-selective nature of participation and older adolescent age of our sample, the generalizability of findings may be limited. Surveys were completed 8 months after the initial stay at home order, and consequently, recall bias may have impacted responses to items inquiring about the start of the pandemic.

Overall, research with larger samples is needed to understand how adolescents and young adults with bingeing behaviors and BED are impacted during times of extreme stress. Due to the changing nature of the pandemic and the increased demand for mental health services, this study suggests that close clinical attention must be given to these individuals.

## Conclusions

The COVID-19 pandemic and subsequent public health measures have adversely impacted the mental health and well-being of many adolescents and young adults. In our sample, almost half reported clinically significant bingeing behaviors. Our study reinforces the importance of not only recognizing our patients’ exposure to COVID-19 but assessing and understanding the potential impact of the pandemic on multiple domains of their lives. It is important to recognize that adolescents and young adults experienced not only an interruption in their daily lives, but also in their identity development and maturity and may require more intensive support from family, schools, and health care providers. Finally, it is critical that medical institutions provide more extensive psychological support for patients who are exhibiting binge-eating behaviors and help them develop adaptive coping strategies in the context of the ongoing COVID-19 pandemic.

## Data Availability

The datasets generated and/or analyzed during the current study are not publicly available due to patient confidentiality and the commitment given to all participants in protecting their identity. Data are available de-identified from the corresponding author on reasonable request and IRB approval.

## Abbreviations

EDs: Eating disorders
BED: Binge eating disorder
BES: Binge eating scale
CEFIS-AYA: COVID-19 Exposure and Family Impact Survey-Adolescent and Young Adult Version

## References

[1] Castellini G, Cassioli E, Rossi E, Innocenti M, Gironi V, Sanfilippo G (2020). The impact of COVID-19 epidemic on eating disorders: a longitudinal observation of pre versus post psychopathological features in a sample of patients with eating disorders and a group of healthy controls. Int J Eat Disord.

[2] Termorshuizen JD, Watson HJ, Thornton LM, Borg S, Flatt RE, MacDermod CM, Harper LE, van Furth EF, Peat CM, Bulik CM (2020). Early impact of COVID-19 on individuals with self-reported eating disorders: a survey of ~ 1000 individuals in the United States and the Netherlands. Int J Eat Disord.

[3] Graell M, Morón-Nozaleda MG, Camarneiro R, Villaseñor A, Yáñez S, Muñoz R (2020). Children and adolescents with eating disorders during COVID-19 confinement: difficulties and future challenges. Eur Eat Disord Rev.

[4] Qian J, Hu Q, Wan Y, Li T, Wu M, Ren Z (2013). Prevalence of eating disorders in the general population: a systematic review. Shanghai Arch Psychiatry.

[5] Kjeldbjerg ML, Clausen L (2021). Prevalence of binge-eating disorder among children and adolescents: a systematic review and meta-analysis. Eur Child Adolesc Psychiatry Publ Online.

[6] Giel KE, Schurr M, Zipfel S, Junne F, Schag K (2021). Eating behaviour and symptom trajectories in patients with a history of binge eating disorder during COVID-19 pandemic. Eur Eat Disord Rev.

[7] Mason TB, Barrington-Trimis J, Leventhal AM (2021). Eating to cope with the COVID-19 pandemic and body weight change in young adults. J Adolesc Health.

[8] Phillipou A, Meyer D, Neill E, Tan EJ, Toh WL, Van Rheenen TE (2020). Eating and exercise behaviors in eating disorders and the general population during the COVID-19 pandemic in Australia: initial results from the COLLATE project. Int J Eat Disord.

[9] Nagata JM, Seligman HK, Weiser SD (2021). Perspective: the convergence of coronavirus disease 2019 (COVID-19) and food insecurity in the United States. Adv Nutr.

[10] Schanzenbach D, Pitts A. Estimates of food insecurity during the COVID-19 crisis: results from the COVID impact survey, week 1 (April 20–26, 2020). Northwestern.edu. https://www.ipr.northwestern.edu/documents/reports/food-insecurity-covid_week2_report-18-may-2020.pdf. Accessed 15 Dec 2021.

[11] Tester JM, Rosas LG, Leung CW (2020). Food insecurity and pediatric obesity: a double whammy in the era of COVID-19. Curr Obes Rep.

[12] Larson N, Laska MN, Neumark-Sztainer D (2020). Food insecurity, diet quality, home food availability, and health risk behaviors among emerging adults: findings from the EAT 2010–2018 Study. Am J Public Health.

[13] Zickgraf HF, Stefano E, Price J, Veldheer S, Rogers A, Rigby A (2019). The relationship between food insecurity and binge and night eating symptoms in prebariatric surgery patients is mediated by depressive symptoms. Surg Obes Relat Dis.

[14] Weissman RS, Bauer S, Thomas JJ (2020). Access to evidence-based care for eating disorders during the COVID-19 crisis. Int J Eat Disord.

[15] Rasmusson G, Lydecker JA, Coffino JA, White MA, Grilo CM (2018). Household food insecurity is associated with binge-eating disorder and obesity. Int J Eat Disord.

[16] Christensen KA, Forbush KT, Richson BN, Thomeczek ML, Perko VL, Bjorlie K (2021). Food insecurity associated with elevated eating disorder symptoms, impairment, and eating disorder diagnoses in an American University student sample before and during the beginning of the COVID-19 pandemic. Int J Eat Disord.

[17] McLaughlin KA, Green JG, Alegría M (2012). Food insecurity and mental disorders in a national sample of U.S. adolescents. J Am Acad Child Adolesc Psychiatry.

[18] Nagata JM, Palar K, Gooding HC, Garber AK, Whittle HJ, Bibbins-Domingo K (2019). Food insecurity is associated with poorer mental health and sleep outcomes in young adults. J Adolesc Health.

[19] Fang D, Thomsen MR, Nayga RM (2021). The association between food insecurity and mental health during the COVID-19 pandemic. BMC Public Health.

[20] Han BB, Purkey E, Davison CM (2022). Food worry and mental health outcomes during the COVID-19 pandemic. BMC Public Health.

[21] Polsky JY, Gilmour H (2020). Food insecurity and mental health during the COVID-19 pandemic. Health Rep.

[22] Gormally J, Black S, Daston S, Rardin D (1982). The assessment of binge eating severity among obese persons. Addict Behav.

[23] Marcus MD, Wing RR, Hopkins J (1988). Obese binge eaters: affect, cognitions, and response to behavioral weight control. J Consult Clin Psychol.

[24] COVID-19 exposure and family impact scales (CEFIS). Healthcaretoolbox.org. https://www.healthcaretoolbox.org/covid19-exposure-family-impact-scale. Accessed 15 Dec 2021.

[25] R: a language and environment for statistical computing. Gbif.org. https://www.gbif.org/tool/81287/r-a-language-and-environment-for-statistical-computing. Accessed 14 Dec 2021

[26] Isomaa R, Isomaa A-L, Marttunen M, Kaltiala-Heino R, Björkqvist K (2010). Psychological distress and risk for eating disorders in subgroups of dieters. Eur Eat Disord Rev.

[27] Mason TB, Lewis RJ (2014). Profiles of binge eating: the interaction of depressive symptoms, eating styles, and body mass index. Eat Disord.

[28] Touyz S, Lacey H, Hay P (2020). Eating disorders in the time of COVID-19. J Eat Disord.

[29] Manasse SM, Schumacher LM, Goldstein SP, Martin GJ, Crosby RD, Juarascio AS (2018). Are individuals with loss-of-control eating more prone to dietary lapse in behavioural weight loss treatment? An ecological momentary assessment study. Eur Eat Disord Rev.

[30] Hoyt LT, Cohen AK, Dull B, Maker Castro E, Yazdani N (2021). “Constant stress has become the new normal”: stress and anxiety inequalities among US college students in the time of COVID-19. J Adolesc Health.

[31] Meherali S, Punjani N, Louie-Poon S, Abdul Rahim K, Das JK, Salam RA (2021). Mental health of children and adolescents amidst COVID-19 and past pandemics: a rapid systematic review. Int J Environ Res Public Health.

[32] Hazzard VM, Loth KA, Hooper L, Becker CB (2020). Food insecurity and eating disorders: a review of emerging evidence. Curr Psychiatry Rep.

[33] Hazzard VM, Hooper L, Larson N, Loth KA, Wall MM, Neumark-Sztainer D (2022). Associations between severe food insecurity and disordered eating behaviors from adolescence to young adulthood: findings from a 10-year longitudinal study. Prev Med.

[34] Coleman-Jensen A, McFall W, Nord M. Food insecurity in households with children: prevalence, severity, and household characteristics, United States Department of Agriculture (USDA)Economic Research Service. 2010–11. 2013 10.22004/ag.econ.262126.

[35] Branley-Bell D, Talbot CV (2020). Exploring the impact of the COVID-19 pandemic and UK lockdown on individuals with experience of eating disorders. J Eat Disord.

[36] Brown S, Opitz M-C, Peebles AI, Sharpe H, Duffy F, Newman E (2021). A qualitative exploration of the impact of COVID-19 on individuals with eating disorders in the UK. Appetite.

[37] Frayn M, Fojtu C, Juarascio A (2021). COVID-19 and binge eating: patient perceptions of eating disorder symptoms, tele-therapy, and treatment implications. Curr Psychol.

[38] Schlegl S, Maier J, Meule A, Voderholzer U (2020). Eating disorders in times of the COVID-19 pandemic-results from an online survey of patients with anorexia nervosa. Int J Eat Disord.

[39] Pedrini L, Meloni S, Lanfredi M, Ferrari C, Geviti A, Cattaneo A (2022). Adolescents' mental health and maladaptive behaviors before the Covid-19 pandemic and 1-year after: analysis of trajectories over time and associated factors. Child Adolesc Psychiatry Ment Health.

